# Characterization of aflatoxin producing *Aspergillus flavus* from food and feed samples

**DOI:** 10.1186/s40064-015-0947-1

**Published:** 2015-04-01

**Authors:** Md Fakruddin, Abhijit Chowdhury, Md Nur Hossain, Monzur Morshed Ahmed

**Affiliations:** Institute of Food Science and Technology (IFST), Bangladesh Council of Scientific and Industrial Research (BCSIR), Dhaka, Bangladesh

**Keywords:** *Aspergillus*, Mycotoxin, Production, Sequencing

## Abstract

*Aspergillus flavus* is one of the major producers of aflatoxin and can contaminate wide range of agricultural commodities either in field or in storage. 15 presumptive *Aspergillus flavus* has been isolated from 30 feed and grain samples. All the isolates were morphologically similar to *Aspergillus flavus* type strains. All the isolates were found to be aflatoxigenic. DNA sequencing of 5.8 s rDNA confirmed all of them to be *Aspergillus flavus*. Only 1 isolate possessed all the seven toxigenic gene (*aflR*, *aflS*, *aflQ*, *aflP*, *aflD*, *aflM*, and *aflO*) while *afl*P & *afl*Q were most prevalent in the isolates. All the isolates possessed at least three toxigenic genes. Toxin producing ability in solid culture media showed that 11 isolates isolates were able to produce both aflatoxin B1 & B2. More than 90% isolates produced aflatoxin B1 ranging 7–22 μg/g of agar. This study alarms us about the potential risks of *Aspergillus flavus* to public health if contaminate agricultural commodities such as grains or raw materials such as poultry feed. Proper harvest and storage management is required to reduce the risk of aflatoxin in feed and grains.

## Introduction

One of the most important ubiquitous fungal species in tropical environments is *Aspergillus flavus* that can be found in soil and other substrates (Powell *et al*. 1994). *Aspergillus flavus* is reported to be associated with many diseases of human, most severe of which is invasive aspergillosis. It can also cause diseases in insects (Campbell, 1994) as well as in crops (such as maize, rice, peanuts etc.). Agricultural products including cereals e.g. maize, wheat, sorghum and by products thereof and variety of oilseeds are major constituents of poultry feed (Okoli *et al*. 2006). Agricultural commodities if contaminated with toxigenic fungi like *A. flavus* producing mycotoxin can be injurious for animals and human health. Production of mycotoxins is species specific; therefore, proper identification and characterization of fungi is of prime importance to develop any prevention strategy (Dawlatana *et al.*^2008^). Different mycotoxins have been reported as contaminant of poultry feed, most important of which are aflatoxins (B1, B2, G1, G2) and Ochratoxin A (OTA) (Gentles *et al*. 1999). Aflatoxins are the most studied group of mycotoxins which apart from producing clinical toxicosis also reduce the resistance to diseases and interfere with vaccine induced immunity in poultry birds (Sharma, 1993). Regular monitoring of toxigenic mycoflora of the agricultural based feeds and foods is an essential pre-requisite for development of strategies to control or prevent mycotoxins exposure of feed animal and human population.

Mycotoxins are fungal secondary metabolites reported to be potentially harmful to animals or humans. Predominant species with aflatoxin production ability include *A. flavus* and *A. parasiticus* (Yu *et al.*^2004^). Of at least 16 structurally close aflatoxin has been detected to date, aflatoxins B1, B2, G1 and G2 are the major four (Goldblatt, 1969). Most studied aflatoxin is aflatoxin B1 as it is identified to be most toxic and potentially hepatocarcinogenic (Bennett and Klich, 2003).

Considerable amount of crop and livestock losses occur due to contamination of toxigenic fungi and mycotoxin. Economic loss increased also due to conducting regulatory programs to prevent such contamination. Feeding livestock and poultry with aflatoxin contaminated feeds can cause death and immune suppression as well as growth reduction. Lowe yields of animals and crops can also be occurred due to aflatoxin contamination (Phillips *et al.*^1996^).

Major aims of the present study were to isolate and characterize aflatoxigenic *Aspergillus flavus* from food and feed samples of Bangladesh.

## Results

### Isolation

**Figure 1:**
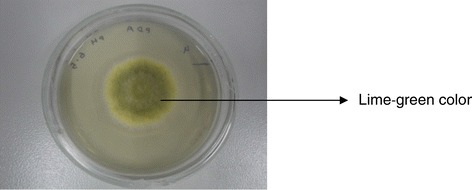
**4 day growth of** ***A. flavus*** **IMS1103 on potato dextrose agar at 25°C.**

15 presumptive *Aspergillus flavus* strains has been isolated from the 30 samples (15 feed and 15 grain sample; grain sample include nuts and pulses; all the samples were collected from local market) analyzed (Figure 1). Total *A. flavus* load in feed samples ranged from 5.6 to 7.1×10^−2^ propagules per gram and in grain samples ranged from 2.8 to 3.8×10^−2^ propagules per gram of grain. Of the 15 isolate, 6 were isolated from poultry feed and 9 were isolated from grain samples. All the isolates were preserved in glycerol broth at −80°C and working cultures were maintained at 4°C.

### Microscopic observation

**Figure 2:**
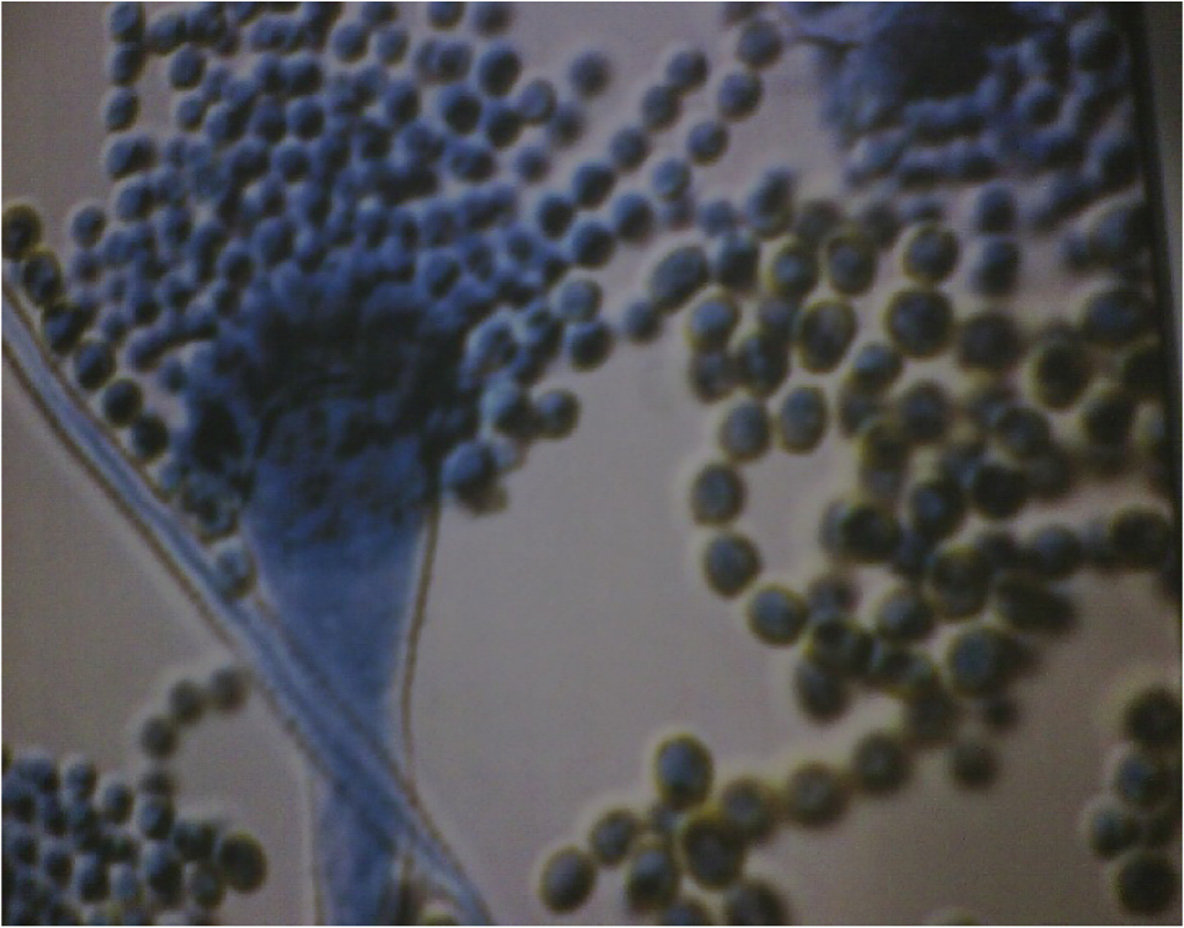
**Photography of isolated** ***Aspergillus flavus*** **strain in the laboratory.**

The distinct colonies were stained on a slide using Lactophenol Cotton Blue. When observed in the microscopic slide, conidiophores was heavy walled, uncolored, coarsely roughened. Vesicles was elongated. Conidia size was around 3-5 μm and appeared as sub-globose and round. Conidia walls showed to be finely rough and in chain orientation. Conidial masses radiate from conidia head. Hyphae was septate, pellicle formations were also observed (Figure 2).

### Screening of aflatoxin production

After 7 days incubation at 25°C in Hara medium, zones of diffusible aflatoxin around the fungal colony was observed under long wave (365 nm) UV light. This indicated that all the isolates have aflatoxin producing capability and thus selected for further studies.

### Identification

DNA sequencing of 5.8 s rDNA of the isolates shows more than 98% resemblance with sequences of *Aspergillus flavus* deposited in database and is thus confirmed as *Aspergillus flavus*.

### Aflatoxin gene profile

In terms of possession of aflatoxin genes, the isolates varied widely. None of the gene was found to be present in all the 15 isolates. Among 15 isolates, aflR was possessed by 11 (73.3%); aflS (aflJ), aflD (nor-1), aflM (ver 1), aflO (omtB) were possessed by 12 (80%) isolates each; aflP (omtA) by 13 (86.7%) and aflQ (ordA) was possessed by 14 (93.3%) isolates. aflP and aflQ were most prevalent genes in the *Aspergillus flavus* isolates (Table 1).

**Table 1:** **Aflatoxigenic gene profile of** ***Aspergillus flavus*** **isolates**

**Isolate no.**	***aflR***	***aflS (aflJ)***	***aflD (nor-1)***	***aflM (ver-1)***	***aflO (omtB)***	***aflP (omtA)***	***aflQ (ordA)***
1	+	-	+	+	+	+	+
2	+	+	-	+	-	+	+
3	-	+	+	+	+	+	+
7	+	-	+	+	+	-	+
21	+	+	-	+	+	+	+
24	+	+	+	+	-	+	+
26	+	+	+	-	+	+	+
29	-	+	+	+	+	+	+
32	+	+	+	+	+	+	-
34	+	-	+	+	+	+	+
35	-	+	+	-	+	+	+
37	-	+	+	+	-	+	+
39	+	+	+	+	+	-	+
40	+	+	-	-	+	+	+
41	+	+	+	+	+	+	+

Five isolate (isolate no-2, 7, 35, 37 and 40) (33.3%) had 5 toxigenic genes each, nine isolate (isolate no- 1, 3, 21, 24, 26, 29, 32, 34, 39) (60%) had 6 toxigenic genes each, and only one isolate (isolate no-41) (6.7%) had all the seven toxigenic genes (Table 1).

### Quantification of aflatoxin produced in Culture media

Aflatoxin producing ability of the isolates was tested on solid agar media, Czapek Dox agar. Four types of aflatoxin production were assayed (B1 & B2) and quantified. Aflatoxin production by the *Aspergillus flavus* isolates in Czapek Dox agar media are showed in Table 2.

**Table 2:** **Production of AFB1 (μg/g agar) by** ***A. flavus*** **isolates on Czapek Dox agar media**

**Strain no**	**Source**	**Aflatoxin production (μg/g agar)**		**Strain no**	**Source**	**Aflatoxin production (μg/g agar)**	
		**AFB1**	**AFB2**			**AFB1**	**AFB2**
1	Feed	11.8	-	29	Grain	25.7	17.6
2	Feed	10.4	5.8	32	Grain	31.6	21.5
3	Feed	14.6	7.1	34	Grain	28.6	19.2
7	Feed	18.2	9.9	35	Grain	33.6	9.3
21	Feed	6.3	-	37	Grain	29.7	11.5
24	Feed	7.8	4.5	39	Grain	21.7	8.9
26	Feed	-	12.3	40	Grain	26.8	16.7
				41	Grain	32.1	-

## Discussion

A group of secondary metabolites produced by members of *Aspergillus* spp. (commonly by *Aspergillus flavus* and *Aspergillus parasiticus*) is known as aflatoxins (Kuiper-Goodman, 1998). These are ubiquitous in nature, associated with the spoilage and toxin production of stored, agricultural commodities (Hedayati et al. 2007). Considerable importance is associated with the presence of aflatoxins in food and feed because of their carcinogenic, mutagenic and teratogenic effects (Begum and Samajpati, 2000). Aflatoxins have been detected in many agricultural products as well as processed products (Bennett and Klich, 2003). The intake limits for humans of about 0.15 ng/kg/day for aflatoxins B1 and 0.20 ng/kg/day for aflatoxins M1 (Kamal et al. 2009). *Aspergillus flavus* are one of the most important producers of aflatoxin. Agricultural commodities can be contaminated with *A. flavus* at any stage of production (in field/during harvest/transport & storage) (Berthier and Valla 1998).

Extensive research work on aflatoxin contamination in agriculture products have been done worldwide, but report on occurrence and identity of toxigenic fungi and level of mycotoxin in different products of Bangladesh is very little. On this context, this study is an effort to investigate distribution of toxigenic fungi in grains and feeds. Permitted level of aflatoxin in Bangladesh is 4 μg/kg, and previous studies conducted in Bangladesh reports that agricultural commodities such as maize, rice etc. are contaminated with 33 to 480 ppb of aflatoxin (Dawlatana et al. 2002). Present study as well as previous reports (Begum et al. 1994; Dawlatana et al. 2002) indicate that grains and feeds of Bangladesh are contaminated with toxigenic fungi and aflatoxin. Grains and feeds are consumed in large quantity in Bangladesh and aflatoxin contamination of these products pose threat to the consumers of Bangladesh.

*Aspergillus flavus* was found to be most predominant species in grains and feed samples which is in accordance with Reddy et al. (2004) who also reported *Aspergillus* species as one of the most common fungus in grains and poultry feed samples. 15 Aspergillus flavus strains was isolated & identified on the basis of cultural characteristics on PDA & microscopic observation. All the isolate produced lime-green colored colony on PDA & characteristic conidia in chain.

Isolates- 1, 7, 21, 24, 26, 32, 34, 35, 39 & 40 are phylogenetically closer to *Aspergillus flavus*. All these strains except Isolate-26 were capable to produce AFB1. 11 isolates (2, 3, 7, 24, 29, 32, 34, 35, 37, 39, 40) were capable to produce both types of toxins (AFB1 & AFB2). *A. flavus* isolates from grain showed more aflatoxin production capability than those from feed. All the isolates possessed at least 5 (out of 7) aflatoxigenic genes. 5 isolate possessed 5 toxigenic gene each; 9 isolate possessed 6 toxigenic gene each and only 1 isolate possessed all the 7 aflatoxigenic gene. Though all aflatoxigenic genes were not detected in most of the isolates while the isolates producing aflatoxin, this discrepancy may be due to inability of the primers used in this study to amplify the genes.

Amount of aflatoxin produced in culture media alarms us about the potential of the isolates to produce high amount of aflatoxin in agricultural commodities. There are no recent surveillance data on the amount of aflatoxin contamination in grains and feeds in Bangladesh. The AFB1 value was higher than the minimum standard institute regulated level in this study. Since grains are one of the main diets of the local individuals and upon consumption of large amount of contaminated grains will create serious consequences. Aflatoxin contamination in poultry feed is also alarming due to its bioaccumulation in poultry that can be subsequently be deleterious for humans. Therefore, the management of storage is very important and improvement in storage conditions for preventing the spoilage and reducing the AFB1 contamination is recommended.

## Conclusion

Aflatoxin contamination in food and feed is a serious risk for public health having long-term health effects. Most of the crops used in culinary in Bangladesh have been reported to be contaminated with aflatoxigenic *Aspergillus flavus* and/or aflatoxins. Grains are stored for long time before distribution which make them vulnerable to aflatoxin contamination. Continuous surveillance should be conducted to detect aflatoxin contaminated crops and contamination level in different food products. Good agricultural management practice should be employed to reduce contamination risk of aflatoxins and aflatoxigenic fungi in agricultural commodities.

## Materials and methods

### Sample collection

15 poultry feed samples and 15 grain samples were collected from different local farms and shops of Dhaka, Bangladesh during February-May 2011. These samples were collected aseptically following standard microbiological protocol.

### Isolation & Identification of aflatoxin producing Aspergillus flavus

Samples were enriched in Buffered Peptone water (BPW) solution on a horizontal shaker for 30 minutes at room temperature. *Aspergillus spp* were identified and isolated in *Aspergillus* differentiation media (ADM) as described by Sreekanth et al. (2011) and Klich (2002).

### Screening of aflatoxin production

Screening of aflatoxin production by the isolates using Hara et al. (1974) media was performed according to Sekar et al. (2008). *A. flavus* was plated on Hara media and incubated at 25°C for 7 days in dark. Diffusible zone of aflatoxin was detected under long wave (365 nm) UV light as blue fluorescent.

### DNA extraction

DNA from the Aspergillus flavus isolates were extracted and quantified according to Fakruddin et al. (2012). Fungal mycelia was collected in sterile saline water and vortexed vigorously with glass beads to crush the mycelial wall and to release the DNA. The mixture was then centrifuged and supernatant collected and DNA extracted from it by phenol/chloroform and ethanol precipitation.

### Identification

Phylogenetic identification on the basis of sequencing of highly variable region of the fungal 5.8S rDNA gene was performed as described in Fakruddin et al. (2013). Amplification of the region was performed by PCR. Primers used in this assay were nu-SSU-0817-5′(TTAGCATGGAATAATRRAATAGGA) and nu-SSU- 1196-3′ (TCTGGACCTGGTGAGTTTCC). Sequencing reactions were carried out using ABI-Prism Big dye terminator cycle sequencing ready reaction kit and the PCR products were purified by a standard protocol. The purified cycle sequenced products were analyzed with an ABI-Prism 310 genetic analyzer. The chromatogram sequencing files were edited using Chromas 2.32. The homology of the 5.8S rDNA gene sequences was checked with the 5.8S rDNA gene sequences of other organisms that had already been submitted to GenBank database using the BLASTN algorithm.

### Detection of aflatoxin genes

Aflatoxin biosynthetic genes (*aflR*, *aflS*, *aflQ*, *aflP*, *aflD*, *aflM*, and *aflO*) in the isolated *Aspergillus flavus* was detected according to Gallo et al. (2012) using primers described in Table 3.

**Table 3:** **Sequences of the nucleotide primers used in this study**

**Primer code**	**Target gene**	**Primer sequences**	**Product size (bp)**
AflR-1for	*aflR*	5′-AAGCTCCGGGATAGCTGTA-3′	1079
AflR-2rev		5′-AGGCCACTAAACCCGAGTA-3′	
AflS-1for	*aflS (aflJ)*	5′-TGAATCCGTACCCTTTGAGG-3′	684
AflS-2rev		5′-GGAATGGGATGGAGATGAGA-3′	
AflD-1for	*aflD (nor-1)*	5′-CACTTAGCCATCACGGTCA-3′	852
AflD-2rev		5′-GAGTTGAGATCCATCCGTG-3′	
AflM-1for	*aflM (ver-1)*	5′-AAGTTAATGGCGGAGACG-3′	470
AflM-2rev		5′-TCTACCTGCTCATCGGTGA-3′	
AflO-1for	*aflO (omtB)*	5′-TCCAGAACAGACGATGTGG-3′	790
AflO-2rev		5′-CGTTGGCTAGAGTTTGAGG-3′	
AflP-1for	*aflP (omtA)*	5′-AGCCCCGAAGACCATAAAC-3′	870
AflP-2rev		5′-CCGAATGTCATGCTCCATC-3′	
AflQ-1for	*aflQ (ordA)*	5′-TCGTCCTTCCATCCTCTTG-3′	757
AflQ-2rev		5′-ATGTGAGTAGCATCGGCATTC-3′	

### Aflatoxin production on agar media

Aflatoxin-producing ability of the isolates was performed by cultivating the fungal strains in Czapek Yeast extract agar medium and extracted and estimated by HPLC (Reddy et al. 2009).

### HPLC analysis of Aflatoxin production

HPLC monitoring of aflatoxin was carried out according to Ben Fredj et al. (2009). Mixture of water: acetic acid: acetonitrile (57:41:2, v/v/v) was used as mobile phase. Flow rate was 1.0 ml/min, injection volume was 100 μl and retention time was 15 min.

## References

[CR1] Begum F, Samajpati N (2000). Mycotoxin production on rice, pulses and oilseeds. Naturwissenschaften.

[CR2] Begum F, Samajpati N, Chatterjee GC (1994). Production and purification of aflatoxin B1 from a selected strain of *Aspergillus flavus* Link on rice. Bang J Sci Indus Res.

[CR3] Ben Fredj SM, Chebil S, Mliki A (2009). Isolation and characterization of ochratoxin A and aflatoxin B1 producing fungi infecting grapevines cultivated in Tunisia. African J Microbiol Res.

[CR4] Bennett JW, Klich M (2003). Mycotoxins. Clin Microbiol Rev.

[CR5] Berthier J, Valla G (1998). Moisissures - mycotoxineset aliments: du risque à la prévention. Revue de Médecine Vétérinaire.

[CR6] Campbell CK, Powell KA, Renwick A, Peberdy JF (1994). Forms of aspergillosis. The genus *Aspergillu*s.

[CR7] Dawlatana M, Coker RD, Nagler MJ, Wild CP, Hassan MS, Blunden G (2002). The occurrence of mycotoxins in key commodities in Bangladesh: surveillance results from 1993 to 1995. J Nat Toxins.

[CR8] Dawlatana M, Shahida S, Rahim M, Hassan MT (2008). Investigation on the occurrence of Ochratoxin A in Maize in Bangladesh. Bang J Sci Indus Res.

[CR9] Fakruddin M, Islam S, Ahmed MM, Chowdhury A, Hoque MM (2012). Development of multiplex PCR (Polymerase Chain Reaction) method for detection of *Salmonella* spp. and *Vibrio parahaemolyticus* from shrimp samples of Bangladesh. Asian J Bio Sci.

[CR10] Fakruddin M, Islam MA, Quayum MA, Ahmed MM, Chowdhury N (2013). Characterization of stress tolerant high potential ethanol producing yeast from agro-industrial waste. Am J Biosci.

[CR11] Gallo A, Stea G, Battilani P, Logrieco AF, Perrone G (2012). Molecular characterization of an *Aspergillus flavus* population isolated from maize during the first outbreak of aflatoxin contamination in Italy. Phytopathologia Mediterranea.

[CR12] Gentles A, Smith EE, Kubena LF, Duffus E, Johnson P, Thompson J, Harvey RB, Edrington TS (1999). Toxicological evaluation of cyclopiazonic acid and Ochratoxin A in broilers. Poult Sci.

[CR13] Goldblatt LA (1969). Aflatoxin.

[CR14] Hara S, Fennell DI, Hesseltine CW (1974). Aflatoxin-producing strains of *Aspergillus flavus* detected by fluorescence of Agar medium under ultraviolet light. Appl Microbiol.

[CR15] Hedayati MT, Pasqualotto AC, Warn PA, Bowyer P, Denning DW (2007). *Aspergillus flavus*: human pathogen, allergen and mycotoxin producer. Microbiol.

[CR16] Kamal ASM, Khair A, Dawlatana M, Hassan MT, Begum F, Rahim M (2009). Evaluation of Aflatoxins and pesticide residues in fresh and different processed mushrooms. Bang J Sci Indus Res.

[CR17] Klich MA (2002). Identification of Common *Aspergillus* Species.

[CR18] Kuiper-Goodman T (1998) In Mycotoxins and Phytotoxins- Developments in Chemistry, Toxicology and Food Safety. Miraglia M, van Edmond H, Brera C, Gilbert J (eds). Fort Collins, CO: Alaken Inc, pp 125

[CR19] Okoli IC, Nweke CU, Okoli CG, Opara MN (2006). Assessment of the mycoflora of commercial poultry feeds sold in the humid tropical environment of Imo State, Nigeria. Int J Environ Sci Tech.

[CR20] Phillips SI, Wareing PW, Dutta A, Panigrahi S, Medlock V (1996). The mycoflora and incidence of mycotoxin, zearalenone and sterigmatocystin in dairy feed and forage samples from Eastern India and Bangladesh. Mycopathologia.

[CR21] Powell KA, Renwick A, Peberdy JF (1994). The genus *Aspergillus*: from taxonomy and genetics to industrial application.

[CR22] Reddy CS, Reddy KRN, Kumar RN, Laha GS, Muralidharan K (2004). Exploration of aflatoxin contamination and its management in rice. Indian J Myco Plant Pathol.

[CR23] Reddy KRN, Saritha P, Reddy CS, Muralidharan K (2009). Aflatoxin B1 producing potential of *Aspergillus flavus* strains isolated from stored rice grains. African J Biotechnol.

[CR24] Sekar P, Yumnam N, Ponmurugan K (2008). Screening and characterization of mycotoxin producing fungi from dried fruits and grains. Adv Biotechnol.

[CR25] Sharma RP (1993). Immunotoxicity of mycotoxins. J Dairy Sciences.

[CR26] Sreekanth D, Sushim GK, Syed A, Khan BM, Ahmad A (2011). Molecular and morphological characterization of a taxol-producing Endophytic fungus, *Gliocladium*sp., from *Taxusbaccata*. Mycobiology.

[CR27] Yu J, Whitelaw CA, Nierman WC, Bhatnagar D, Cleveland TE (2004). *Aspergillus flavus* expressed sequence tags for identification of genes with putative roles in aflatoxin contamination of crops. FEMS Microbiol Letters.

